# A novel homozygous mutation in POLR3A gene causing 4H syndrome: a case report

**DOI:** 10.1186/s12887-018-1108-9

**Published:** 2018-04-04

**Authors:** Vishal V. Tewari, Ritu Mehta, C. M. Sreedhar, Kunal Tewari, Akbar Mohammad, Neerja Gupta, Sheffali Gulati, Madhulika Kabra

**Affiliations:** 1 Departments of Pediatrics, Army Hospital (Referral & Research), New Delhi, 110010 India; 2 Department of Pathology, All India Institute of Medical Sciences, New Delhi, India; 3 Department of Radiology, Army Hospital (Referral & Research), New Delhi, India; 4 Department of Anesthesia, Base Hospital, New Delhi, India; 5 Department of Pediatrics, All India Institute of Medical Sciences, New Delhi, India

**Keywords:** Congenital hypomyelinating leukodystrophy, 4H syndrome, POLR3A gene, Hypodontia, Hypogonadotropic hypogonadism

## Abstract

**Background:**

4H syndrome is a congenital hypomyelinating leukodystrophy characterized by hypodontia, hypomyelination and hypogonadotropic hypogonadism belonging to the Pol III-related leukodystrophies which arise due to mutations in the POLR3A or POLR3B gene. The clinical presentation is of neurodevelopmental delay or regression with ataxia, dystonia, nystagmus, delayed deciduous dentition and abnormal order of eruption of teeth. MRI brain shows a characteristic hypomyelination pattern. Several mutations have been described in the implicated genes but there are no reports on mutations seen in patients from India.

**Case presentation:**

We report a 1½ year old girl, only child of a non-consanguinous couple who presented with delayed developmental milestones and delayed dentition. On physical examination she had downward slanting palpebral fissures, low set ears, smooth philtrum, hypodontia, prominent body hair and clitoromegaly. There was prominent horizontal nystagmus, hypertonia of both upper and lower limbs, exaggerated deep tendon jerks and flexor planter response. She had not attained complete head control and required support to sit. She showed absent waves on brainstem evoked response audiometry and her fundus examination showed bilateral optic atrophy with prolongation of P100 latencies on visual evoked potentials. MRI Brain showed hyperintensity of entire white matter with involvement of the internal and external capsule, frontal deep white matter and corpus callosum. Her karyotype was 46 XX and her endocrinal profile was unremarkable. Clinical exome sequencing identified an unreported mutation in the POLR3A gene. The same mutation was identified by Sanger sequencing in heterozygous state in both parents. The child is being managed with physiotherapy and developmental therapy. She has been provided with hearing aids and started on speech therapy. Parents were provided anticipatory guidance and genetic counselling about autosomal recessive nature of inheritance, risk of recurrence and need for follow-up.

**Conclusion:**

4H syndrome is a rare congenital hypomyelinating leukodystrophy inherited as an autosomal recessive disorder due to mutations in the POLR3A and POLR3B gene. Delay or regression of milestones, abnormalities in dentition and endocrinal perturbations are its hallmark. A novel mutation in the POLR3A gene resulting in amino acid substitution of arginine for glutamine at codon 808 (p.R808Q) was detected in exon 18 in our case.

**Electronic supplementary material:**

The online version of this article (10.1186/s12887-018-1108-9) contains supplementary material, which is available to authorized users.

## Background

The 4H syndrome (4HS) is a white matter neuroregression syndrome of recent coinage and description which is characterized by hypomyelination, hypodontia, and hypogonadotropic hypogonadism. The entity was first described by Timmons et al. as a progressive hypomyelinating leukodystrophy of unknown origin characterized by central and peripheral hypomyelination, hypogonadotropic hypogonadism of pituitary origin with hypodontia in 4 unrelated adults between 20 to 30 year ages and was suggested to have an autosomal recessive or a de novo dominant inheritance. These patients had remained normal up to the prepubertal age [1]. It is now understood that 4H syndrome belongs to a group of Pol III-related leukodystrophies characterized by a combination of three major clinical findings i.e. motor dysfunction, abnormal dentition and hypogonadotropic hypogonadism resulting in several clinical phenotypes. Apart from the 4H syndrome the Pol III-related leukodystrophies includes four other syndromes of ataxia, delayed dentition, and hypomyelination (ADDH), tremor-ataxia with central hypomyelination (TACH), leukodystrophy with oligodontia (LO) and hypomyelination with cerebellar atrophy and hypoplasia of the corpus callosum (HCAHC) [2]. Pol III-related leukodystrophies are inherited in an autosomal recessive manner and result from mutations in POLR3A gene on chromosome 10q22 or the POLR3B genes on chromosome 12q23. These genes are responsible for encoding the two largest subunits of RNA polymerase III (Pol III) which is a multi-subunit complex composed of 17 subunits. It has been hypothesized that Pol III is crucial for tRNA synthesis and these mutations lead to abnormality in tRNA levels in the brain. There are 14 different mutations in the POLR3A gene which have been described in 19 patients of French-Canadian, Caucasian and Syrian ethnicity. The mutations were spread throughout the gene, and there were no obvious genotype/phenotype correlations [3]. There are two earlier reports of 4H syndrome from India published in medical literature. However mutation studies were not reported in these cases [4, 5]. In this case report we highlight the presentation, evaluation, management and genetic counselling of a 1 ½ year old girl child with 4H syndrome with a novel homozygous mutation in the POLR3A gene.

## Case presentation

**Fig. 1:**
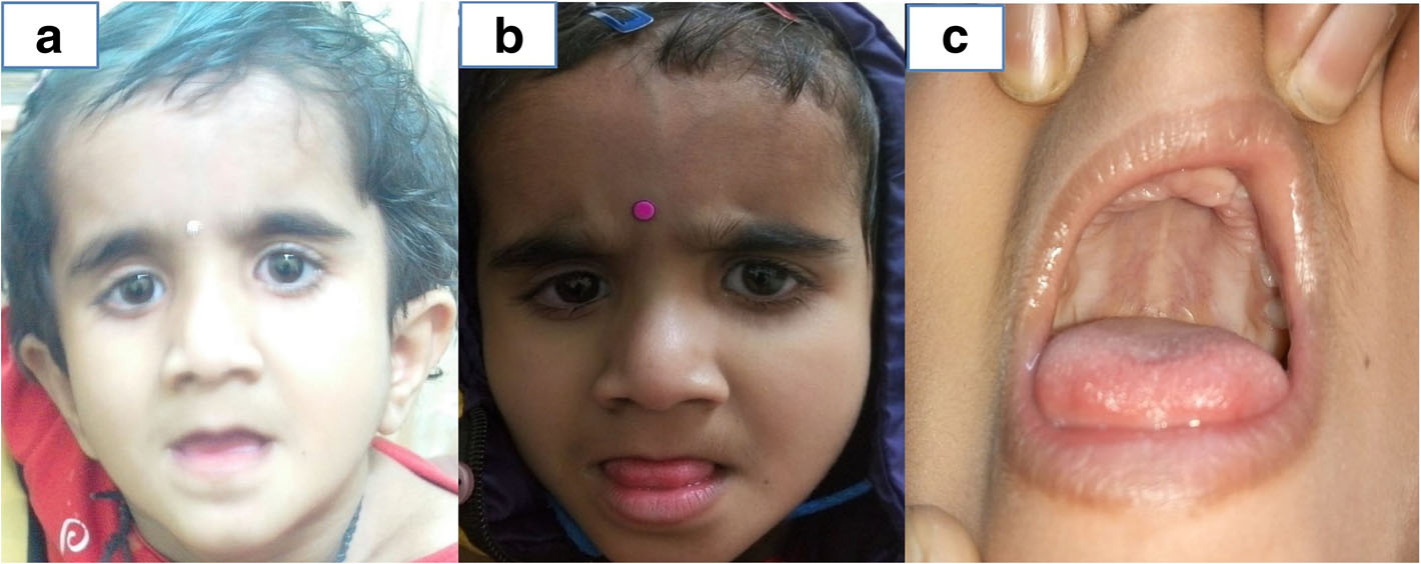
**a** Photograph of the child aged 2 years showing downward slant of palpebral fissures, low set ears, smooth philtrum and thin upper lip. **b** and **c** Photograph of the child aged 3 years showing facial features as earlier and hypodontia

**Fig. 2:**
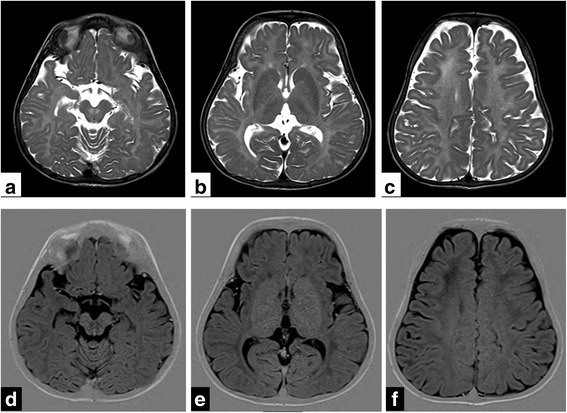
**a**, **b**, **c**, **d**, **e**, **f** Matched TSE T2W axial images (**a** to **c**) and IR T1W axial images (**d** to **f**) of the brain show diffuse signal alteration in the entire cerebral white matter including the internal and external capsules and the pre-aqueductal region, appearing hyperintense on T2WI and hypointense on matched T1WI. There is diffuse sulcal prominence indicative of cerebral atrophy

**Fig. 3:**
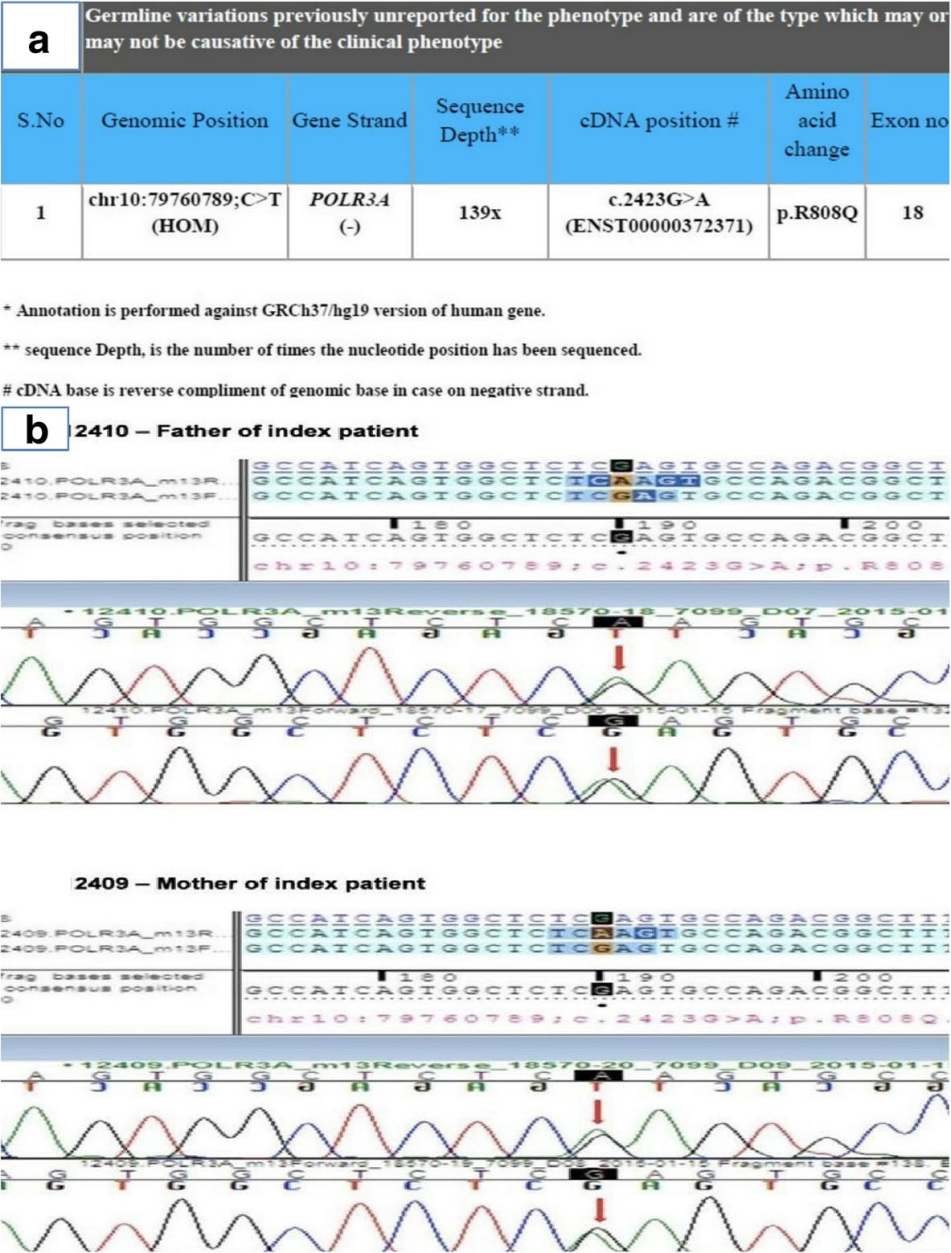
**a** Clinical exome sequencing report of the index case showing a previously unreported mutation c.2423G > A in the POLR3A gene producing an amino acid change p.R808Q. **b** Electropherograms of the mother and father of the index case. Sequence chromatogram and alignment to the reference sequence showing variation detected in the parents

A 1½ year old girl child, only child of a non-consanguineous couple presented to the pediatric department of our institute with complaints of delayed developmental milestones and delayed appearance of primary dentition. The child had been delivered at term by normal vaginal delivery following an uneventful antenatal and intrapartum period with a birth weight of 2.3 kg. There was history of a previous abortion at 9 weeks period of gestation. As a neonate and young infant she had shown poor neurobehaviour with inability to breast feed, weak cry and poor activity. The child had shown delay in attainment of milestones in all spheres of development since early infancy. By the end of 1 year age the child had not attained stable head holding, was unable to turn from prone to supine, had an immature grasp and had nystagmus involving both eyes evident on lateral gaze noted since 5 months age. Parents also noted that the infant had delayed dentition and had no teeth till one year of age. There was no history of seizures. She had been fed top milk with cup and spoon till 6 months age and age-appropriate complementary feeding using traditional home foods had been introduced thereafter. At presentation her weight was 7.2 kg (< 5^th^ centile for age), length was 75 cm (< 5^th^ centile for age) and occipitofrontal circumference was 48.2 cm (< 5^th^ centile). On head to toe examination the child showed facial dysmorphism in the form of downward slant of palpebral fissures, low set ears, smooth philtrum, and thin lips with hypodontia (Fig 1a, b, c). She had broad thumbs, prominent body hair and clitoromegaly. On systemic examination there was prominent horizontal nystagmus, hypertonia of both upper and lower limbs with exaggerated deep tendon jerks but down going planter reflex in both limbs. She had not attained complete head control and required support to sit. The child made good eye contact and was interactive and responsive and made sounds to indicate hunger. She expressed interest in toys by pointing towards them and looked at mothers face for approval. Her motor developmental age corresponded to 6 months. There was no palpable hepatosplenomegaly or any other palpable lump. There was no gonad palpable in the labia. She had poor response to sound at soft levels on behavioural observation audiometry (BOA). Hearing evaluation by transient evoked oto-acoustic emission (TEOAE) showed ‘refer’ responses bilaterally and no waveforms were discerned on brainstem evoked response audiometry (BERA) even at 110 dB. Examination of fundus showed bilateral optic disc pallor and optic atrophy with no pigmentary retinopathy. Visual evoked potentials (VEP) showed prolongation of P100 latencies on both sides while the electroencephalogram (EEG) was normal. X-ray lumbosacral spine showed squared iliac wings with decreasing interpedicular distance caudally from lumbar vertebra 1 to 5 (LV1 to LV5) with flattened acetabular roof and posterior scalloping of vertebrae. USG abdomen showed uterus of size 5.4 × 13.4 × 16.5 mm and normal adenexa. MRI Brain showed diffuse signal alterations in the entire cerebral white matter with involvement of the internal capsule, external capsule, frontal deep white matter and corpus callosum appearing hyperintense on T2 weighted and hypointense on T1 weighted images consistent with hypomyelination (Fig 2a, b, c, d, e, f). Her thyroid profile was normal. Other parameters in her hormonal profile were a 17-OH progesterone value of 0.16 ng/ml (normal < 1 ng/ml), LH value of 0.14 IU/L (normal < 1–3.3 IU/L), FSH 2.17 IU/L (normal < 1–7.1 IU/L), estradiol 44.22 pg/ml (normal < 20–53 pg/ml) and GH value of 6.4 mcg/L (normal < 10 mcg/L). Her karyotype was done in view of the clitoromegaly and was 46XX. Multiplex ligation-dependent probe amplification (MLPA) analysis of 1p36 region for copy number loss/gain was done using microdeletion kit P064-B1 (MRC-Holland) which did not show any microdeletions or microduplications. The findings of craniofacial dysmorphism with developmental delay, hypodontia, clitoromegaly, hypomyelination on MRI Brain and the need for genetic counselling of the family prompted the decision to investigate the child by Targeted sequencing analysis. DNA extracted from peripheral blood was used to perform targeted gene capture using Numblegen SeqCap EZ choice XL (Roche, USA) custom capture kit. Kapa library preparation kit was used to prepare whole genome libraries followed by target enrichment with biotinylated probes. These libraries were sequenced to mean > 80-100× coverage on illumina sequencing platform. The sequences obtained were aligned to human reference genome (GRCh37/hg 19) using BWA program and analysed using Picard and GATK-Lite tool kit. Clinically relevant mutations were annotated using published variants in literature and a set of variant databases including ClinVar, OMIM, GWAS, HGMD and SwissVar. The targeted sequencing analysis was performed at a reference laboratory (MedGenome Labs Bangalore India). An unreported homozygous missense variation in exon 18 of POLR3Agene (chr10:79760789;C > T; c.2423G > A) resulting in amino acid substitution of Arginine for Glutamine at codon 808(p.R808Q;ENDT00000372371) was detected (Fig 3a). This POLR3A variant is not reported in the 1000 genomes database and is predicted to be damaging by PolyPhen, LRT and Mutation Taster. The region is conserved across species. On this basis, the POLR3A variation was classified as a possibly significant variant. Based on the clinical presentation, MRI brain pattern and result of targeted Next Generation Sequencing she was diagnosed as a case of 4H syndrome occurring due to a novel mutation in the POLR3A gene. The child was enrolled into physiotherapy and developmental therapy programme. She was provided with bilateral hearing aids and started on speech therapy. Parents were counselled about the risk of seizure and seizure precautions in the child, need for close follow-up, the autosomal recessive nature of inheritance, risk of recurrence and sequencing for this variation in them. Both parents were tested using Sanger sequencing and were found to be heterozygous carriers of the same mutation detected in the child (Fig 3b). The child is on regular follow-up and has become ambulant with assistance (Additional file 1) and continues to be seizure free.

## Discussion and conclusions

Congenital hypomyelinating disorders are a heterogeneous group of CNS leukoencephalopathies amongst which the Pol III-related leukodystrophies present with varying combinations of motor dysfunction, abnormal dentition, and hypogonadotropic hypogonadism and share features on brain MRI. Hypomyelinating leukodystrophies are identified once two MRIs done at least six months apart after the age of one year show no significant improvement in the myelination pattern. These syndromes have recently been recognised to result from mutations involving POLR3A, POLR3B genes and more recently the POLR1C gene and are inherited as autosomal recessive disorders [6]. There are five phenotypes emanating from these mutations i.e. 4H syndrome, ADDH, TACH, LO and HCAHC as enumerated earlier with no obvious genotype/phenotype correlation seen [3]. The age of onset shows correlation with the severity of the disorder and the phenotype with an earlier onset correlating with a greater severity while POLR3A mutations lead to a more severely affected phenotype.

The 4H leukodystrophy shows both neurological and non-neurological manifestations. Delayed attainment of motor milestones or regression of previously achieved milestones with delayed walking, ataxic gait and dystonia progressing to wheelchair dependence is noted. Inability to walk by 2 years age indicates poor prognosis in terms of attainment of independent ambulation. Ataxia is the main hindrance to walking while dystonia is not seen commonly. Children with earlier onset show more ataxia, nystagmus and tremors. Prominent cerebellar signs with varying degrees of pyramidal signs are seen. Seizures are infrequent and optic atrophy is also not consistently seen. Deterioration of speech and swallowing occurs with time while comprehension of speech and non-verbal communication remain preserved till late. Intellectual deterioration is gradually progressive with mild to moderate mental subnormality seen in all cases [7, 8]. The non-neurological manifestations include dental abnormalities and endocrinal perturbations. The primary dentition is delayed and an abnormal order of eruption of the deciduous dentition is seen with the deciduous molar erupting before the incisors. Presence of natal teeth, misshapen and missing teeth and hypodontia are the other abnormalities noted [7]. Hypogonadotropic hypogonadism results in delayed onset of puberty due to impaired secretion of gonadotropic releasing hormone (GnRH), defect in GnRH receptor or GnRH bioactivity. These patients usually have abnormal baseline FSH and LH levels and there is absence of response to pituitary stimulation with GnRH. GH deficiency has been recognised and has been reported as a cause for clinical deterioration in later years [8–10].

MRI Brain findings of hypomyelination, hypointensities in the posterior limb of internal capsule (PLIC), ventrolateral thalamus and dentate nucleus on T2 weighted images have been reported. Myelination of the pyramidal tracts within the PLIC and the optic radiation is seen while supratentorial atrophy and thinning of corpus callosum is seen much later reflecting generalized atrophy and white matter loss. Cerebellar atrophy is seen in virtually all the cases with POLR3B mutations while it is absent or mild in patients with POLR3A mutation. MRI pattern recognition allows identification of the patient without dental or endocrinal abnormalities [7, 8, 11]. The exact pathogenesis of this disorder is not completely understood. On histopathologic examination a lack of myelin and reduced numbers of oligodendrocytes with the degree of neuronal loss in proportion to the involvement of myelin is seen. Oligodendroglial loss is most severe in the centrum semiovale, while the oligodendroglia are most preserved in the white matter surrounding small capillaries, venules and arterioles [7, 12].

The protein encoded by the POLR3A gene is the catalytic component of Pol III, which synthesizes small RNAs such as 5S rRNA and tRNAs. It plays a key role in sensing and limiting infection by intracellular bacteria and DNA viruses and acts as nuclear and cytosolic DNA sensor involved in innate immune response by detecting foreign DNA. These Pol III mutations cause abnormal tRNA transcription leading to cytoplasmic synthesis alteration and relative abundance of certain tRNA in the CNS [13] as compared to other organs in the body and accounts for the neurological involvement in 4H syndrome. Recently a subset of patients have been identified with clinical and radiological manifestations of 4H syndrome but no identifiable mutation in the POLR3A and POLR3B genes. In these cases recessive mutations have been found in POLR1C, a gene encoding for a subunit common to POLR1 and POLR3, which has thus far been known only to be associated with autosomal recessive Treacher Collins syndrome [6]. Large exonic deletions or duplications in the POLR3A and POLR3B genes have also been reported to result in 4H syndrome [14].

Early studies on patients with the 4H syndrome described clinical and neuroradiographic findings and identified its occurrence in patients of Indian [4], Somalian [15], Italian [16], Syrian [17], Swiss [18], German [18, 19], Canadian [19] and Turkish [19] ancestry. The age at presentation was highly variable from 18 months [16] to 17 years [15] with the commonest reported age being 11–13 years [17–19]. Genome wide screening using short tandem repeats (STR) and single nucleotide polymorphism (SNP) markers in some of these patients identified the genetic locus to 10q22 and a search for candidate genes was undertaken [20]. A recent large cross sectional multi-national study identified 105 mutation confirmed cases with 43 patients having a mutation in the POLR3A gene. This study showed that patients of European descent mostly had mutations in the POLR3B gene barring those of French Canadian ancestry who carried the c.2015G.A mutation in the POLR3A gene. Patients of Mediterranean ancestry also carried mutations mostly in POLR3A. POLR3A gene mutations resulted in an earlier age of onset, a more rapid neurological decline and a shorter life expectancy. However a clear genotype-phenotype correlation could not be established [8]. In our case a hitherto unreported homozygous missense mutation (chr10: 79760789;C > T; c.2423G > A) resulting in the amino acid substitution of arginine for glutamine at codon 808 (p.R808Q) was detected in exon 18 of POLR3A gene. Our patient also had an early presentation with progressive neurodevelopmental regression. It is possible that this mutation is associated with a severe phenotype, although this can only be confirmed after more cases are diagnosed and mutations identified. Exclusion of 1p36 deletion syndrome in our case by MLPA was done in view of the facial dysmorphism with hypodontia. Evaluation for presence of other copy number variants (CNVs) associated with microdeletions or microduplications which may account for the facial dysmorphism would require chromosomal microarray (CMA) analysis. The diagnosis of the disorder and management of the case requires a multidisciplinary approach. Genetic counselling should be offered to every patient.

In conclusion 4H syndrome is a congenital hypomyelinating leukodystrophy characterized by neurodevelopmental regression, hypodontia and hypogonadotropic hypogonadism. There is wide variation in the presentation but disorders due to POLR3A gene mutations are more severe. In this case report we report a novel homozygous missense mutation (chr10: 79760789; C > T; c.2423G > A) resulting in the amino acid substitution of arginine for glutamine at codon 808 (p.R808Q) detected in exon 18 of POLR3A gene. MRI Brain can help in making the diagnosis. A multidisciplinary approach is needed for the diagnosis and management.

## Additional file

Additional file 1: Video clip of the child. Child at the age of 5 year 10 months showing ataxic gait, nystagmus, tremors and requiring assistance during ambulation. (MP4 50864 kb)

## Abbreviations

ADDH: Ataxia, delayed dentition, and hypomyelination
BERA: Brainstem evoked response audiometry
BOA: Behavioural observation audiometry
DNA: Deoxyribonucleic acid
EEG: Electroencephalogram
FSH: Follicle stimulating hormone
GH: Growth hormone
GnRH: Gonadotropic releasing hormone
HCAHC: Hypomyelination with cerebellar atrophy and hypoplasia of the corpus callosum
LH: Luteinizing hormone
LO: Leukodystrophy with oligodontia
MLPA: Multiplex ligation dependent probe amplification
PLIC: Posterior limb of internal capsule
rRNA: Ribosomal ribonucleic acid
TACH: Tremor-ataxia with central hypomyelination
TEOAE: Transient evoked oto-acoustic emission
tRNA: Transcription ribonucleic acid
VEP: Visual evoked potential
